# Supplementary material for: A Critical View of the Application of the APEX Software (Aqueous Photochemistry of Environmentally-Occurring Xenobiotics) to Predict Photoreaction Kinetics in Surface Freshwaters
Source: Molecules. 2019 Dec 18;25(1):9. doi: 10.3390/molecules25010009 (PMC7017383; doi:10.3390/molecules25010009)
Supplement: Supplementary file 1 [file molecules-25-00009-s001.zip › 3.2.4_gcc-4.4.0.zip!/3.2.4_gcc-4.4.0/3.2.4_gcc-4.4.0/doc/octave/html/interpreter/Opening-and-Closing-Files.html]

Opening and Closing Files - Untitled


Next: Simple Output,
Up: C-Style I/O Functions


---

#### 14.2.1 Opening and Closing Files

When reading data from a file it must be opened for reading first, and
likewise when writing to a file. The `fopen` function returns a
pointer to an open file that is ready to be read or written. Once all
data has been read from or written to the opened file it should be closed.
The `fclose` function does this. The following code illustrates
the basic pattern for writing to a file, but a very similar pattern is
used when reading a file.

```
     filename = "myfile.txt";
     fid = fopen (filename, "w");
     # Do the actual I/O here...
     fclose (fid);
```

— Built-in Function: [fid, msg] = **fopen** (name, mode, arch)  
— Built-in Function: fid\_list = **fopen** ("all")  
— Built-in Function: [file, mode, arch] = **fopen** (fid)  
> The first form of the `fopen` function opens the named file with
> the specified mode (read-write, read-only, etc.) and architecture
> interpretation (IEEE big endian, IEEE little endian, etc.), and returns
> an integer value that may be used to refer to the file later. If an
> error occurs, fid is set to −1 and msg contains the
> corresponding system error message. The mode is a one or two
> character string that specifies whether the file is to be opened for
> reading, writing, or both.
>
> The second form of the `fopen` function returns a vector of file ids
> corresponding to all the currently open files, excluding the
> `stdin`, `stdout`, and `stderr` streams.
>
> The third form of the `fopen` function returns information about the
> open file given its file id.
>
> For example,
>
> ```
>           myfile = fopen ("splat.dat", "r", "ieee-le");
> ```
>
> opens the file splat.dat for reading. If necessary, binary
> numeric values will be read assuming they are stored in IEEE format with
> the least significant bit first, and then converted to the native
> representation.
>
> Opening a file that is already open simply opens it again and returns a
> separate file id. It is not an error to open a file several times,
> though writing to the same file through several different file ids may
> produce unexpected results.
>
> The possible values mode may have are
>
> r: Open a file for reading. w: Open a file for writing. The previous contents are discarded. a: Open or create a file for writing at the end of the file. r+: Open an existing file for reading and writing. w+: Open a file for reading or writing. The previous contents are discarded. a+: Open or create a file for reading or writing at the end of the file.
>
> Append a "t" to the mode string to open the file in text mode or a
> "b" to open in binary mode. On Windows and Macintosh systems, text
> mode reading and writing automatically converts linefeeds to the
> appropriate line end character for the system (carriage-return linefeed
> on Windows, carriage-return on Macintosh). The default if no mode is
> specified is binary mode.
>
> Additionally, you may append a "z" to the mode string to open a
> gzipped file for reading or writing. For this to be successful, you
> must also open the file in binary mode.
>
> The parameter arch is a string specifying the default data format
> for the file. Valid values for arch are:
>
> native
> The format of the current machine (this is the default).
>
> ieee-be
> IEEE big endian format.
>
> ieee-le
> IEEE little endian format.
>
> vaxd
> VAX D floating format.
>
> vaxg
> VAX G floating format.
>
> cray
> Cray floating format.
>
> however, conversions are currently only supported for native
> ieee-be, and ieee-le formats.
>
>
>
> **See also:** fclose, fgets, fputs, fread, fseek, ferror, fprintf, fscanf, ftell, fwrite.

— Built-in Function: **fclose** (fid)  
> Closes the specified file. If successful, `fclose` returns 0,
> otherwise, it returns -1.
>
>
>
> **See also:** fopen, fseek, ftell.
